# Transcription factor and microRNA regulation in androgen-dependent and -independent prostate cancer cells

**DOI:** 10.1186/1471-2164-9-S2-S22

**Published:** 2008-09-16

**Authors:** Guohua Wang, Yadong Wang, Weixing Feng, Xin Wang, Jack Y Yang, Yuming Zhao, Yue Wang, Yunlong Liu

**Affiliations:** 1 School of Computer Science and Technology, Harbin Institute of Technology, Harbin, Heilongjiang 150001, PR China; 2 Division of Biostatistics Department of Medicine, Indiana University School of Medicine, Indianapolis, IN 46202, USA; 3 Center for Computational Biology and Bioinformatics, Indiana University School of Medicine, Indianapolis, IN 46202, USA; 4 Center for Medical Genomics, Indiana University School of Medicine, Indianapolis, IN 46202, USA; 5 Department of Surgery, Indiana University School of Medicine, Indianapolis, IN 46202, USA; 6 College of Automation, Harbin Engineering University, Harbin, Heilongjiang 150001, PR China

## Abstract

**Background:**

Prostate cancer is one of the leading causes of cancer death in men. Androgen ablation, the most commonly-used therapy for progressive prostate cancer, is ineffective once the cancer cells become androgen-independent. The regulatory mechanisms that cause this transition (from androgen-dependent to androgen-independent) remain unknown. In this study, based on the microarray data comparing global gene expression patterns in the prostate tissue between androgen-dependent and -independent prostate cancer patients, we indentify a set of transcription factors and microRNAs that potentially cause such difference, using a model-based computational approach.

**Results:**

From 335 position weight matrices in the TRANSFAC database and 564 microRNAs in the microRNA registry, our model identify 5 transcription factors and 7 microRNAs to be potentially responsible for the level of androgen dependency. Of these transcription factors and microRNAs, the estimated function of all the 5 transcription factors are predicted to be inhibiting transcription in androgen-independent samples comparing with the dependent ones. Six out of 7 microRNAs, however, demonstrated stimulatory effects. We also find that the expression levels of three predicted transcription factors, including AP-1, STAT3 (signal transducers and activators of transcription 3), and DBP (albumin D-box) are significantly different between androgen-dependent and -independent patients. In addition, microRNA microarray data from other studies confirm that several predicted microRNAs, including miR-21, miR-135a, and miR-135b, demonstrate differential expression in prostate cancer cells, comparing with normal tissues.

**Conclusion:**

We present a model-based computational approach to identify transcription factors and microRNAs influencing the progression of androgen-dependent prostate cancer to androgen-independent prostate cancer. This result suggests that the capability of transcription factors to initiate transcription and microRNAs to facilitate mRNA degradation are both decreased in androgen-independent prostate cancer. The proposed model-based approach indicates that considering combinatorial effects of transcription factors and microRNAs in a unified model provides additional transcriptional and post-transcriptional regulatory mechanisms on global gene expression in the prostate cancer with different hormone-dependency.

## Background

Prostate cancer is the second leading cause of cancer death in males in the United States [1]. When androgen ablation therapy, an commonly-used treatment protocol, becomes ineffective, prostate tumors progress from androgen dependent (AD) to androgen independent (AI) stage [2]. In the past decade, many studies were conducted to investigate the mechanism that causes the transition of hormone dependency in prostate cancer [3,4], including low throughput experiments, such as Western blot, real-time PCR, and Northern blot [5,6], and high-throughput studies including microarray experiments [7-9]. Several transcription factors, including AP-1 [7], NF*κ*B[10], and EGR (early growth response factor) [9] etc, were reported to be related to prostate cancer progression. From these experiments, important molecular mechanisms were identified to contribute to the cancer development, including androgen amplification, promiscuous binding, outlaw pathway, bypass pathway, and androgen receptor coregulators [3,4]. Despite these discoveries, however, the complete mechanism of hormone dependency in prostate cancer regulation remains unclear. Such mechanism is further complicated with the recent discovery of microRNA, a class of non-coding RNAs that regulate gene expression in the post-transcriptional level.

MicroRNAs bind to the 3'-untranslated region (3'-UTR) of target transcripts to regulate gene expression by either inhibiting translation or promoting mRNA degradation [11]. Accumulating evidence indicates that microRNAs play critical roles in multiple biological processes, including cell cycle control, cell growth and differentiation, apoptosis, and embryo development [12-15]. Importantly, strong links were established between microRNA deregulation and the occurrences of human diseases, in particular cancer. Genome-wide association studies demonstrated that many human microRNA genes locate at genomic regions linked to cancer [16,17]. Moreover, a recent study found that the absolute expression levels of many microRNAs were reduced significantly in tumors [18]. It was reported that 45 microRNAs differentially expressed in prostate cancer samples comparing with normal tissues [19], including miR-125b, which plays important roles in inducing androgen-independent growth of prostate cancer cells [20].

Similar to transcription factors, microRNAs regulate gene expression in a combinatorial fashion, i.e., individual microRNA can regulate multiple genes, and individual gene can be regulated by multiple microRNAs [21,22]. Based on this principle, we previously developed a model-based approach, *MotifModeler *[23], to identify *de novo *transcription factor and microRNA binding sites from array-derived gene expression data. In this study, we modify the previous approach by focusing on a set of biologically-known transcription factor and microRNA binding sites documented in the TRANSFAC database [24] and microRNA registry [25]. This modification allows direct interpretation of the predicted results.

We apply this model on the microarray data that measure the differences in global gene expression levels in androgen-dependent and androgen-independent prostate tissues [7]. Our model identifies 5 transcription factors and 7 microRNAs that potentially contribute to such differences. The biological functions of predicted transcription factors and microRNAs are further reassured through various bioinformatics analysis.

## Results

### Data set description

In order to investigate the molecular mechanisms underlying the progression of androgen-independent prostate cancer, microarray experiment was conducted in an earlier study, which reported that 239 genes were differentially expressed (p < 0.005) between primary prostate tumors from 10 untreated androgen-dependent and 10 androgen-independent prostate carcinoma patients. In this study, we focus on these 239 differentially expression genes, which include 92 genes and 147 genes are over-expressed and under-expressed in androgen-independent samples, respectively. The original microarray data are retrieved from Gene Expression Omnibus (GEO) database (GEO number: GSE9545).

### Computational identification of functional transcription factors and microRNAs

In order to understand potential transcriptional and post-transcriptional mechanisms that cause the differences in gene expression in AD and AI samples, we develop a computational procedure to identify transcription factors and microRNAs that potentially result in the expressional changes of hundreds of genes. This algorithm is an extension of *MotifModeler*[23], a procedure we previously developed to identify *de novo cis*-acting DNA elements from array-derived gene expression data. In this study, in stead of identifying potential binding sites of transcription factors and microRNAs from all the potential DNA elements of a fixed size (such as hexamers), we focus our investigation on the biologically known transcription factors documented in TRANSFAC database and microRNAs documented in microRNA registry. Among 741 position weight matrices (PWMs) documented in TRANSFAC database [24], 459 PWMs represent binding sites of transcription factors in human, mouse, or rat genome. We further reduce our searching space on the 335 PWMs, where mRNA expression levels of at least one of their binding proteins can be reliably detected in at least 10% of the samples (called "present" using MAS5 algorithm in the original microarray experiment). For microRNA prediction, we use all the 564 microRNA in the microRNA registry [25,26] (miRBase, v.10.1).

In order to identify the functional transcription factors and microRNAs that potentially cause the differences in gene expression between androgen-dependent and -independent samples, we construct a matrix that contains 239 rows representing differentially-expressed genes and 899 columns representing 335 PWMs and 564 microRNAs, respectively. Each element in the matrix denotes a score that describes the binding potential of the corresponding transcription factor or microRNA on the promoter or 3'-UTR of the corresponding gene. The goal of our modelling is to identify a subset of the 899 columns that best describe the expression level differences of the 239 genes.

For each potential transcription factor and microRNA, our procedure calculates a fitness score (GEC: gene expression contributing score) by assessing how well its occurrences in the promoter or 3'-UTR correlate with the expression level difference, in the context of combinatorial regulation, and a functional score (*TF *or *MI*) that evaluates its potential function on the global gene expression difference. A positive and negative functional score implies that its occurrence in the gene regulatory region contributes to the global gene over- and under-expression in the androgen-independent samples comparing with the -dependent samples, respectively.

**Figure 1:**
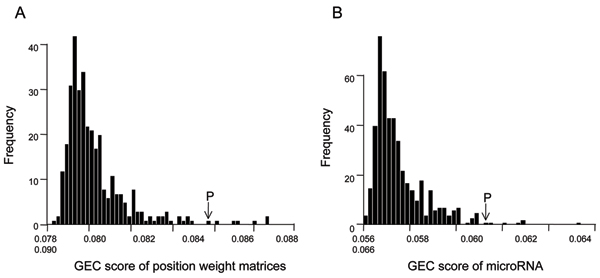
**The histogram of GEC score for known position weight matrices and microRNA binding sites**. (A) Histogram of GEC scores of 335 known PWMs documented in the TRANSFAC database. (B) Histogram of GEC scores of 564 microRNAs.

Fig 1 shows the histograms of GEC scores of all the transcription factors and microRNAs, where a larger GEC score implies a more significant contribution to the differences of gene expression between two prostate tumor groups. In order to distinguish the functional transcription factors and microRNAs from the nonfunctional ones, we only consider the candidates whose GEC scores are larger than mean + 3 × standard deviation as functional regulators (the cutoff is indicated by the arrow in Figure 1). Based on this criterion, 7 PWMs, corresponding to 5 unique transcription factor binding sites (Table 1), and 7 microRNAs with highest GEC score are selected (Table 2). Interestingly, the identified transcription factors and microRNAs demonstrate significant bias on their estimated functional levels. All the 7 PWMs that represent transcription factor binding sites are predicted to contribute to the under-expressions in the AI samples than in AD samples (*TF *< 0), while 6 out of 7 selected microRNAs show opposite functions (*MI *> 0). Considering the fact that microRNAs bind to complementary sites of 3'-UTR to induce RNA degradation, the positive *MI *values can be translated to the decreased capabilities to trigger RNA degradation in androgen-independent prostate tumors.

**Table 1:** GEC scores and predicted function levels (*TF*) of top 7 selected position weight matrices

**Index**	**ID**	**Name**	**PWM Description**	**GEC**	**TF**
1	M00199↓	AP-1	AP-1 binding site	0.0886	-7.61E+05
2	M00225↓	STAT3	signal transducer and activator of transcription 3	0.0885	-6.48E+05
3	M00926↓	AP-1	AP-1 binding site	0.0878	-1.35E+06
4	M00495	Bach1	BTB and CNC homolog 1	0.0870	-5.91E+05
5	M00925↓	AP-1	AP-1 binding site	0.0870	-1.97E+05
6	M00982	KROX	early growth response	0.0860	-6.86E+05
7	M00624↓	DBP	albumin D-box	0.0857	-6.47E+05

↓: the expression level of the transcription factor binding to the predicted binding site is down-regulated in expression array.

**Table 2:** GEC scores and predicted function levels (*MI*) of top 7 selected microRNAs.

**Index**	**Name**	**Mature miRNA sequence**	**GEC**	**MI**
1	hsa-miR-144	u**ACAGUAUA**gaugauguacu	0.0651	8.84E+05
2	hsa-miR-135b	u**AUGGCUUU**ucauuccuauguga	0.0627	6.45E+05
3	hsa-miR-135a	u**AUGGCUUU**uuauuccuauguga	0.0626	5.86E+05
4	hsa-miR-654	u**GGUGGGCC**gcagaacaugugc	0.0625	-5.23E+05
5	hsa-miR-448	u**UGCAUAUG**uaggaugucccau	0.0619	6.35E+05
6	hsa-miR-155	u**UAAUGCUA**aucgugauaggggu	0.0613	5.71E+05
7	hsa-miR-21	u**AGCUUAUC**agacugauguuga	0.0611	5.65E+05

Seed sequences are highlighted by capital letter.

### Expression levels of predicted transcription factors and microRNAs

We further examine the mRNA expression levels of transcription factors that potentially bind on the predicted PWMs. We find that 4 transcription factor genes, JUNB, JUND, STAT3, DBP, whose products bind to 3 out of 5 predicted transcription factor binding sites (AP-1, STAT3, and DBP) are under-expressed in the AI samples in the microarray experiment (indicated by ↓ in table 1). This result provides important evidence that the functional differences of these factors between AI and AD samples are potentially driven by the expressional changes in the transcription level.

In order to evaluate the biological relevance of the predicted microRNAs, we examine their expression profiles in 6 different cancer types, published by an independent study that includes prostate, lung, breast, colon, gastric, and pancreatic cancers, using microRNA microarray technology (Table 3) [19]. Five out of 7 predicted microRNAs are included in the microRNA microarray design. Among them, 3 microRNAs, hsa-miR-135a, hsa-miR-135b, hsa-miR-21 are differentially expressed in prostate tumor comparing with normal samples. Interestingly, hsa-miR-21 is ubiquitously differentially expressed in all six cancer types comparing with normal tissues, while hsa-miR-135a and hsa-miR-135b are specific to prostate tumors. Although no significant expressional difference is detected for hsa-miR-155 between prostate tumors and normal tissues, its signal can be reliably detected in the microRNA microarray (or present) in prostate cancer. In addition, this microRNA is differentially expressed in four other cancer types, including lung, beast, colon, and pancreatic cancers, which suggests its potential roles in regulating tumorigenesis.

**Table 3:** Expression profiles of the predicted microRNAs in six cancer types (prostate, lung, breast, colon, gastric, and pancreatic cancers)

**Index**	**Name**	**Prostate**	**Lung**	**Breast**	**Colon**	**Gastric**	**Pancreatic**
1	hsa-miR-144	Absent					
2	hsa-miR-135b	√					
3	hsa-miR-135a	√					
4	hsa-miR-654	Not present on the array					
5	hsa-miR-448	Not present on the array					
6	hsa-miR-155	Present	√	√	√		√
7	hsa-miR-21	√	√	√	√	√	√

√: the expression level of microRNA is significant different comparing with normal cell.

### Ingenuity pathway analysis

**Figure 2:**
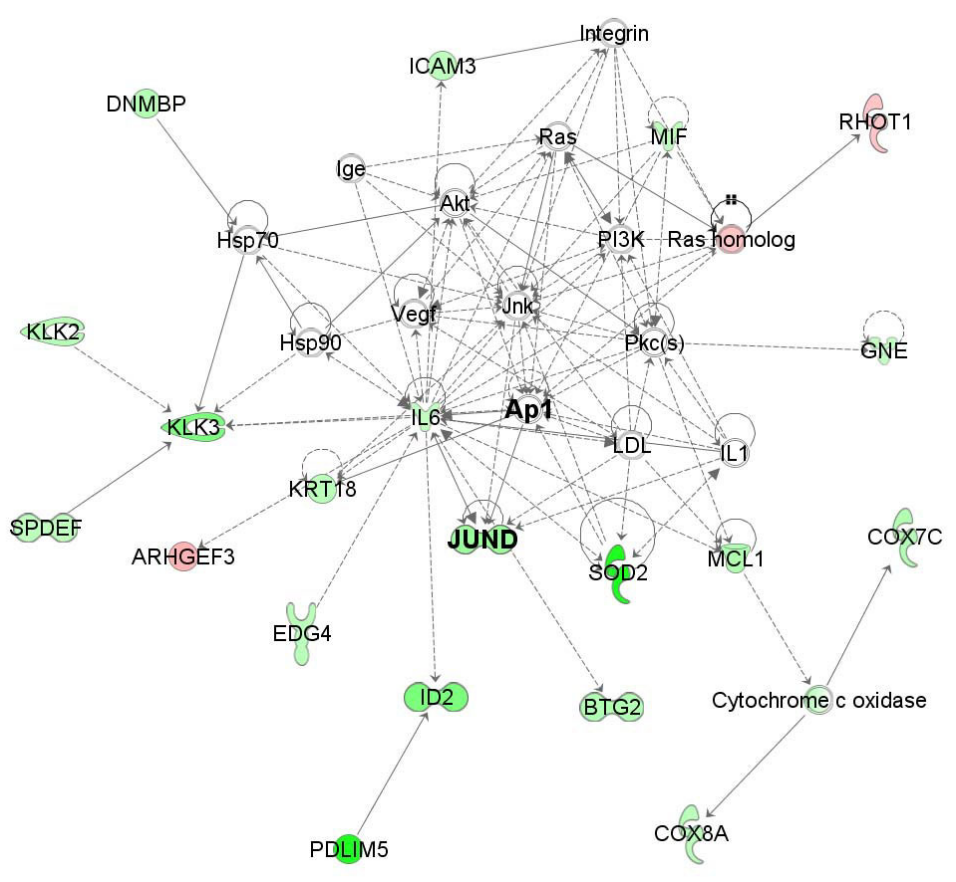
**Ingenuity pathway analysis on AP-1 related gene network**. Green and red nodes represent repressed and induced genes in androgen-independent prostate samples comparing androgen-dependent prostate samples, respectively.

**Figure 3:**
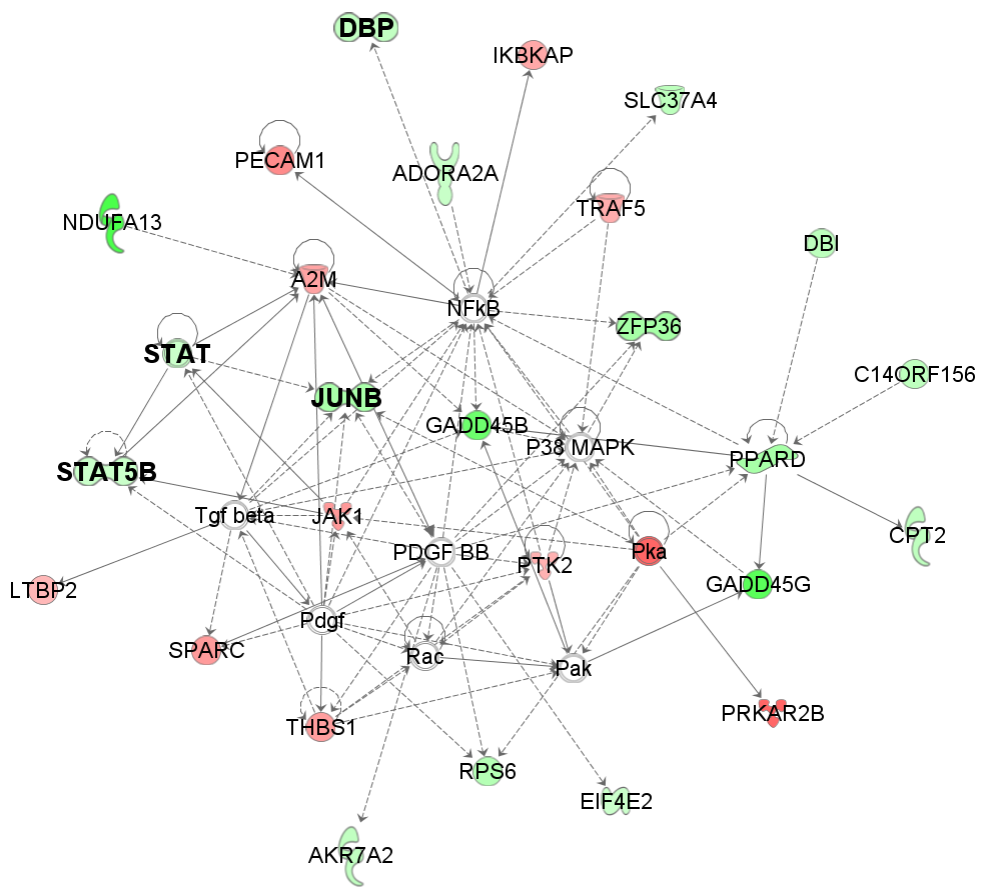
**Ingenuity pathway analysis on NF*κ*B related gene network**. Green and red nodes represent repressed and induced genes in androgen-independent prostate samples comparing androgen-dependent prostate samples, respectively.

Interaction networks among genes differentially expressed between androgen-independent and -dependent tumors are identified using Ingenuity pathway analysis (Fig. 2 and 3). Transcription factors binding on 2 predicted binding sites, AP-1 and STAT, are identified in the enriched networks. AP-1, a protein complex of FOS and JUN, appears at the centre of the network, in which 19 down-regulated genes and 3 up-regulated genes are related. This result is consistent with model prediction, where all the three AP-1-related binding sites are predicted to contribute to the under- expression in androgen-independent samples (*TF *< 0, Table 1).

### Clinical relevance of identified transcription factors

**Figure 4:**
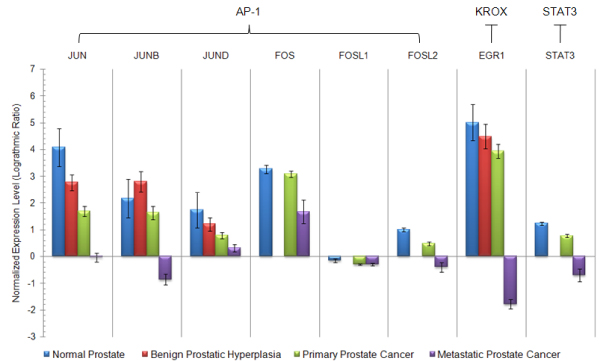
**Expression pattern of predicted transcription factors in different stages of prostate abnormality**. Expression profiles of JUN, JUNB, JUND, EGR are from the same study in Oncomine database, which classifies prostate abnormality into four stages, normal prostate, benign prostatic hyperplasia, primary prostate cancer, and metastatic prostate cancer. FOS, FOSL1, FOSL2, STAT3 are from other two studies both of which include three subtypes, including normal prostate, and primary and metastatic prostate cancer. JUN, JUNB, JUND, FOS, FOSL1, FOSL2 bind to AP-1 binding site, EGR1 binds to KROX binding site, STAT3 binds to STAT3 binding site.

Using Oncomine database, we examine the expression patterns of identified transcription factors in different types of prostate tissues, including normal prostate, benign prostatic hyperplasia, primary prostate tumors and metastatic prostate tumors. Expression levels of eight transcription factors targeting on 3 identified binding sites can be retrieved from Oncomine, where six proteins, JUN, JUNB, JUND, FOS, FOSL1, and FOSL2 bind on AP-1 site, EGR1 (early growth response element 1) binds on KROX site, and STAT3 (signal transducer and activators of transcription 3) binds on STAT3 site. It turns out that expression levels of all the 8 transcription factors have significantly negative correlation with the severity of the disease, which means that with the deterioration of prostate cancer, the gene expression levels of these transcription factors decrease (Fig. 4 and Table 4).

**Table 4:** Expression profiles of the predicted transcription factor in the Oncomine database

**PWM**	**Gene Symbol**	**Genbank ID**	**Correlation**	**P-value**	**Study**
AP-1	JUN	NM_002228	-0.62	2.1E-11	Dhanasekaran_Prostate
	JUNB	NM_002229	-0.57	1.5E-9	Dhanasekaran_Prostate
	JUND	NM_005354	-0.36	2.6E-4	Dhanasekaran_Prostate
	FOS	NM_005252	-0.30	2.0E-3	Lapointe_Prostate
	FOSL1	NM_005438	-0.42	7.0E-3	Vanaja_Prostate
	FOSL2	NM_005253	-0.63	3.9E-13	Lapointe_Prostate
KROX	EGR1	NM_001964	-0.69	3.8E-15	Dhanasekaran_Prostate
STAT3	STAT3	NM_213662	-0.68	1.1E-16	Lapointe_Prostate

### Combinatorial regulation

**Figure 5:**
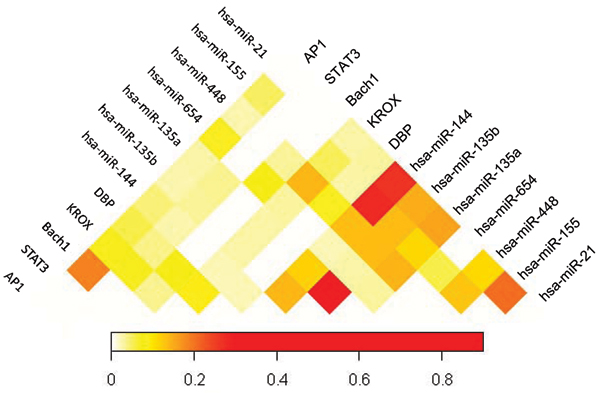
Heatmap of the percentage of target genes that contain co-occurrent transcription factors or microRNAs.

In order to evaluate the combinatorial effects of transcriptional and post-transcriptional regulation that result in the differences in gene expression between AI and AD samples, co-occurrences of any pair of predicted transcription factors or microRNAs in the promoter or 3'-UTR of the 239 differentially expressed genes are examined. Figure 5 shows the percentage of common genes regulated by two transcription factors or microRNAs among the total genes targeted by the same factor pair. Fisher's exact test is used to assess the significance of the co-occurrences of two factors targeting a common gene, and false discovery rate (FDR) is calculated by correcting the p-value with multiple hypotheses. Since hsa-miR-135a and hsa-miR-135b have identical seed and similar sequences, the percentage of predicted commonly-regulated genes is as high as 90%. This number, however, is potentially caused by the artefact that the microRNA-target prediction algorithm being used (PITA) is incapable to distinguish their target genes, and therefore excluded from further discussion. Eleven pairs of transcription factors or microRNAs are found to significantly co-exist in regulating common genes (FDR < 20%, Table 5). Among them, 9 pairs are between microRNAs, 1 pair is between transcription factors (AP-1 and Bach1), and 1 pair are between transcription factor (KROX) and microRNA (hsa-miR-448).

**Table 5:** Transcription factors and microRNAs with common target genes

**Index**	**Factor 1**	**Factor 2**	**Fisher's p-value**	**FDR**
1	hsa-miR-144	hsa-miR-155	0.0%	0%
2	hsa-miR-144	hsa-miR-21	0.0%	0%
3	hsa-miR-155	hsa-miR-21	0.1%	2%
4	AP1	Bach1	0.1%	2%
5	hsa-miR-135a	hsa-miR-21	0.8%	10%
6	hsa-miR-135b	hsa-miR-21	1.3%	14%
7	hsa-miR-135a	hsa-miR-448	1.6%	15%
8	KROX	hsa-miR-448	2.0%	16%
9	hsa-miR-135b	hsa-miR-144	2.2%	16%
10	hsa-miR-135b	hsa-miR-448	2.5%	16%
11	hsa-miR-135b	hsa-miR-155	2.5%	16%

## Discussion

In this study, using known position weight matrices (PWMs) of transcription factors, documented in the TRANSFAC database, and microRNA-target gene prediction, we simultaneously identify the most influential transcription factors and microRNAs that potentially cause the differences in global gene expression profile between androgen-independent and androgen-dependent patients. Focusing on 239 differentially expressed genes between AI and AD samples, our model predicted 5 transcription factors (with 7 different PWMs) and 7 microRNAs that potentially cause gene expression differences. Interestingly, all the 7 PWMs that represent binding sites of 5 transcription factors are predicted to contribute to the decreased levels of gene expression in AI samples, while 6 out of 7 microRNAs are predicted to have opposite effect, i.e., contribute to the increased level of gene expression in AI samples. One potential interpretation is that in androgen-independent prostate tumors, the capability of transcription factors to initiate transcription and microRNAs to degradate mRNA are both repressed.

Many of identified transcription factors are known to be related to the prostate cancer development. AP-1, a transcription factor that consists of dimers of the c-Fos, c-Jun, ATF and JDP families, interacts with DNA through leucine zipper (bZIP) domains. It was reported that physiologically-elevated concentrations of androgens cause prolonged AP-1 DNA binding activity in androgen-responsive prostate carcinoma cell line (LNCaP cells), through induced production of reactive oxygen species. Such effect was not observed in androgen-independent cells (DU145) [27]. It was also reported that AP-1 interacts with androgen receptor both physically and functionally in regulating gene expression [28]. Besides androgen receptors, various evidences suggest that nuclear receptor AP-1 also cross-talks with signal transduction regulations of a large amount of hormone receptors, including estrogen receptor [29], glucocorticoid receptor [30], retinoic acid receptor [31], vitamin D receptor [32], thyroid hormone receptor [33], and so on. In this study, AP-1 is predicted to be an inhibitor in androgen-independent prostate tumors. This prediction is further confirmed with the expression level decrease of JUNB and JUND genes, and suggests that the reduction of AP-1 activity in androgen-independent samples is partially from transcriptional level. Binding site (KROX) of another androgen receptor cofactor, EGR1 (early growth receptor 1), is predicted to contribute to the decreased expression levels in AI samples. Previous report indicated that EGR1 binds to the androgen receptor in prostate carcinoma cells [34]. Over-expression and knockdown of EGR1 enhances and inhibits AR-mediated transactivation and signalling, respectively. Expression levels of EGR1 do not demonstrate significant difference between AI and AD samples, which suggests such functional variation may be induced in post-transcriptional level. The direct correlation between androgen response in prostate cancer and BACH1 is not well understood. This helicase-like factor, however, is known to contribute to DNA repair by interacting with BRCA1 gene [35], which may function as an androgen receptor coregulator and play positive roles in androgen-induced cell death in prostate cancer cells [36]. Abnormal functional levels of BRCA1 in prostate cancer may also cause activation or inhibition of STAT3 (signal transducer and activator of transcription 3) [37], which is predicted by our computational model. In addition, STAT3 is known to induce AR-mediated gene activation in prostate carcinoma via IL-6 signalling pathways [38,39]. D-box binding protein (DBP), a proline and acidic-rich (PAR) protein family member, forms heterdimeric complex with hepatic leukemia factor (HLF) in regulating gene expression [40]. Although the function of DBP on androgen dependency in prostate cancer is not known, we do observe differential expression of DBP between AI and AD prostate tumors.

Besides available biological knowledge, further bioinformatics analysis also supports the potential functions of predicted transcription factors. Ingenuity pathway analysis demonstrates strong link between differentially expressed genes and predicted factors including STAT3, JUNB, JUNC and AP1 (Fig. 2 and 3). In addition, expression profiles of JUN family, FOS family, EGR1, STAT3 monotonically decrease with the deterioration of prostate cancer development, according to the Oncomine database. These clinical evidences are accordant with the predicted functions of identified transcription factors and expression differences in microarray data.

Ingenuity pathway analysis clearly indicates that another nuclear transcription factor, NF*κ*B is closely connected to many differentially expressed genes (Fig. 3). This factor, however, is not predicted by our model. Further investigation suggests that the expression levels of two members in the NF*κ*B gene families, NF*κ*B1 and NF*κ*B2, do not express in either androgen-independent or -dependent prostate tumors, based on the initial microarray experiment. Therefore, it is less likely that NF*κ*B will pose significant influence on the global gene expression differences.

Comparing with transcription factors, functions of individual microRNA on cancer development are much less understood. In recent years, many studies used microRNA expression profiles to classify human cancers and observed that expression levels of many microRNAs were under-expressed in cancers [18]. It becomes clear that as oncogenes and tumor suppressors, microRNAs pose important function in cancer development. Among the 7 identified microRNAs, 6 microRNAs are predicted to contribute to the induced gene expression in androgen-independent prostate tumor. This observation may be caused by lowered expression or reduced function of these microRNAs in androgen-independent prostate samples. Demonstrated in Table 3, most predicted microRNAs are differentially expressed in prostate cancer cells comparing with normal tissues, among which, hsa-miR-155 and hsa-miR-21 are observed in many cancer types [41]. Together with other published reports, prediction of our model supports the hypothesis that differentially expressed microRNAs may contribute to the progression of prostate tumor.

How microRNAs regulate gene expression remains a challenging problem. Based on microRNA target prediction [22,42] and transcription factor binding site database [24], several groups focus on coordinated gene regulation by extracting network motif from pairs of microRNAs and transcription factors [43,44]. The uniqueness of our approach is to integrate the transcription factor and microRNA target prediction into functional data, i.e., array-derived gene expression measurements. This allows identifying functional transcription factors and microRNAs in regulating global gene expression pattern in response to certain biological perturbation or in two different biological conditions. Different from *MotifModeler *[23], a computational approach we developed previously to focus on predicting *de novo cis*-acting DNA elements of a fixed length, the current methodology highlights biologically known information, such as position weight matrices of transcription factors documented in the TRANSFAC database and microRNAs in microRNA registry. This strategy allows direct interpretation of the predicted results, and prompts biological experiments in testing the proposed hypothesis. Despite these advantages, if the goal of the study is to identify *de novo *binding sites, the original *MotifModeler *algorithm remains an appropriate choice.

## Conclusion

We present a model-based computational approach to indentify transcription factors and microRNAs that influence the progression of androgen-dependent prostate cancer to androgen-independent prostate cancer. All the 5 transcription factor binding sites are inhibitory in androgen-independent samples comparing to the androgen-dependent ones. Six out of 7 microRNAs are stimulatory. This result suggests that the capability of transcription factors to initiate transcription and microRNAs to degradate mRNA are both decreased in androgen-independent prostate cancer. The proposed model-based approach indicates that considering combinatorial effects of transcription factors and microRNAs in a unified model provides additional transcriptional and post-transcriptional regulatory mechanisms on global gene expression in the prostate cancer with different hormone-dependency.

## Methods

### Biological system

To identify the important transcription factors and microRNAs which influence the prostate cancer after androgen ablation therapy, we download the gene expression profile from Gene Expression Omnibus Database (GEO number: GSE2443), where Affymetrix Human Genome U133A GeneChip was used to access the global gene expression patterns in 10 androgen-independent prostate tumor biopsies and 10 androgen-dependent prostate tumors.

### Promoter sequence, 3'-UTR sequences and microRNA sequences

Human RefSeq transcript annotation (hg17 genome assembly) is downloaded from the UCSC Genome Browser [45]. For each differentially expressed gene we extract 3'-UTR sequences and promoter sequences up to 1000 bp upstream of transcription start site. Human mature microRNA sequences are downloaded from microRNA registry [25,26] (miRBase, v.10.1).

### Transcription factor and microRNA target prediction

Position weight matrices (PWMs) in the TRANSFAC database are used to predict the transcription factor target genes. For each TF-target gene pair, a series of similarity scores are calculated by scanning the PWM of the transcription factor along the promoter sequences of target gene.

(1) $$S_{ic}=\log_{2}\left(\frac{d_{ic}\sqrt{N_{t}}+b_{ic}}{\sum_{i=A}^{T}d_{ic}\sqrt{N_{t}}+\sum_{i=A}^{T}b_{ic}}/d_{ic}\right)$$

where *N*_*t *_is total number of sample sequences while deriving the PWM in the TRANSFAC database; *d*_*ic *_is the distribute of the *i*-th nucleotide (*i *= A, C, G or T) in the human genome (30% for A and T, and 20% for G and C); *b*_*ic *_is the number of real counts of the *i*-th nucleotide in the *c*-th position in the PWM. For each PWM, we select top 2,000 matching positions with the highest similarity scores in the promoter regions genome-wide as potential TF-target sites.

We adopt PITA algorithm[46] to predict the microRNA-target relationship. While predicting microRNA targets, PITA considers the differences between the energy gained by binding of the microRNA to the transcript target and the energy required to make the target region accessible for microRNA binding. Similar as transcription factor target identification, for each microRNA, top 2,000 microRNA-target interactions with lowest energy difference are selected as candidate microRNA binding sites.

### Transcription factor and microRNA selection procedure

In order to describe the correlation between differences in genes expression levels and the matching scores of transcription factors and microRNAs in the promoter and 3'-UTR, a linear mathematical model is established:

(2) *G*_*K *_= *S*_*K *× *N *_*TF*_*N *_- *E*_*K *× *M *_*MI*_*M*_

where, *G*_*K *_represents logarithmic ratio of observed mRNA expression levels in the AI group comparing to AD group; *S*_*K *× *N *_is the sum of matching score of the *N*-th PWM (Eq. 1) in the promoter region of the *K*-th gene; *E*_*K *× *M *_is the sum of microRNA-target interaction energy (PITA calculation) of the *M*-th microRNAs in the 3'-UTR of the *K*-th gene. In this calculation, only the top 2,000 matching positions genome-wide with highest TF similarity score or lowest microRNA-target interaction energy are considered as transcription factor or microRNA binding loci, respectively. In eqm 2, *TF *and *MI *represent functional levels of transcription factors and microRNAs, respectively. Both of these two numbers are not measurable, and therefore will be estimated using the following iterative procedure.

Similar as *MotifModeler *selection procedure, in each iteration, we randomly pick *N*_*random *_PWMs and *M*_*random *_microRNAs as candidate regulators, and use least-squares method to estimate the functional levels of selected candidate. Since a smaller model error indicates a better selection, a gene expression contributing score (GEC) is assigned to each selected candidate using the following formulation:

(3) $$GEC=\frac{1}{{\left(||G_{K}-(S_{K\times N_{random}}T{\tilde{F}}_{N_{random}}-E_{K\times M_{random}}M{\tilde{I}}_{M_{random}})|{|}^{2}\right)}^{\alpha }}$$

where, $T\tilde{F}$ and $M\tilde{I}$ are estimated functional levels of transcription factors and microRNAs, respectively; *α *is a power factor that influences the effect of single selections (*α *> 1).

In the present study, the procedure to select PWMs and microRNAs can be described as: randomly pick 10 PWMs and 10 microRNAs; estimate *TF *and *MI *using least-squares method; calculate the predicted model error and the current gene expression contributing score (GEC) of each PWM and microRNA; add the current GEC score to the cumulative gene expression contributing score; add PWM function levels (*TF*) and microRNA function levels (*MI*) to the cumulative functional levels of each PWM and microRNA. We repeat this procedure 2 million times; the correlation of GEC score of each 1 million calculation is larger than 0.95. The transcription factors and microRNAs whose GEC scores are larger than mean + 3 × standard deviation are considered as functional regulators. All the programs are written using R 2.6.0 .

### Correlation of predicted transcription factors to clinical gene expression profiles in prostate cancer

In order to investigate the clinical impact of predicted transcription factors in prostate cancer development, Oncomine database is used to examine the expression profiles of all the transcription factors that bind on the predicted binding sites in normal prostate, primary prostate cancer and metastatic prostate cancer.

### Correlation of predicted microRNAs to microRNA expression profiles in cancer samples

We download the microRNA microarray data associated with six tumors from ArrayExpress database [19] (accession number E-TABM-23 for breast cancer, E-TABM-46 for colon cancer, E-TABM-47 for lung cancer, E-TABM-48 for pancreatic cancer, E-TABM-49 for prostate cancer, and E-TABM-50 for gastric cancer). MicroRNAs are defined as present if they are expressed in at least 90% of the samples. The microRNA microarray analysis is conducted following the procedure described in ref [19].

## Competing interests

The authors declare that they have no competing interests.

## Authors' contributions

GW, YW and YL contributed to the design of the study. GW and YL designed and performed the computational modelling and drafted the manuscript. XW, WF, JYY, YZ, and YW participated in coordination, discussions related to result interpretation and revision of the manuscript. All the authors read and approved the final manuscript.
